# Cytokinin delays dark-induced senescence in rice by maintaining the chlorophyll cycle and photosynthetic complexes

**DOI:** 10.1093/jxb/erv575

**Published:** 2016-01-29

**Authors:** Sai Krishna Talla, Madhusmita Panigrahy, Saivishnupriya Kappara, P Nirosha, Sarla Neelamraju, Rajeshwari Ramanan

**Affiliations:** ^1^Centre for Cellular and Molecular Biology, Hyderabad, India; ^2^Directorate of Rice Research, Rajendra Nagar, Hyderabad, India

**Keywords:** Chlorophyll cycle, cytokinin, dark-induced senescence, hydroxymethyl chlorophyll, pigment–protein complex, rice, stay green.

## Abstract

The phytohormone cytokinin (CK) is known to delay senescence in plants. We studied the effect of a CK analog, 6-benzyl adenine (BA), on rice leaves to understand the possible mechanism by which CK delays senescence in a drought- and heat-tolerant rice cultivar Nagina22 (N22) using dark-induced senescence (DIS) as a surrogate for natural senescence of leaves. Leaves of *N22-H-dgl162*, a stay-green mutant of N22, and BA-treated N22 showed retention of chlorophyll (Chl) pigments, maintenance of the Chl *a*/*b* ratio, and delay in reduction of both photochemical efficiency and rate of oxygen evolution during DIS. HPLC analysis showed accumulation of 7-hydroxymethyl chlorophyll (HmChl) during DIS, and the kinetics of its accumulation correlated with progression of senescence. Transcriptome analysis revealed that several plastid-localized genes, specifically those associated with photosystem II (PSII), showed higher transcript levels in BA-treated N22 and the stay-green mutant leaves compared with naturally senescing N22 leaves. Real-time PCR analyses showed that genes coding for enzymes associated with Chl *a*/*b* interconversion and proteins associated with light-harvesting complexes maintained higher transcript levels up to 72h of DIS following BA treatment. The pigment–protein complexes analyzed by green gel remained intact in both *N22-H-dgl162* and BA-treated N22 leaves even after 96h of DIS. Thus, CK delays senescence by accumulation of HmChl and up-regulating genes in the Chl cycle, thereby maintaining the Chl *a*/*b* ratio. Also, CK treatment retains higher transcript levels of PSII-related genes, resulting in the stability of photosynthetic pigment complexes and functional stay-greenness in rice.

## Highlight

Cytokinin induces functional stay-greenness by maintaining the Chl *a/b* ratios and the stability of photosynthetic complexes during dark-induced senescence in rice.

## Introduction

In higher plants, photosynthesis is the process by which light energy from the sun is converted to chemical energy. Chlorophylls (Chls), associated with two pigment–protein complexes, namely photosystem I and II (PSI and PSII), located in the thylakoid membranes of the chloroplast, play a central role in this process. Chl *a* and Chl *b*, the main constituents of the photosynthetic apparatus, are identical molecules except for the side chain at C7, which is a methyl group in the former and a formyl group in the latter (Rüdiger, 2002). Chl *a* is synthesized from glutamate by several steps and is converted to Chl *b* via an intermediate, 7-hydroxymethyl chlorophyll (HmChl), in a cyclic reaction called the Chl cycle, which is well conserved across all land plant species and is important for photosynthesis by green leaves (Tanaka and Tanaka, 2007). The primary energy conversion reactions of photosynthesis are executed in the reaction center (RC), a complex of several proteins, pigments, and cofactors, which, along with the light-harvesting complexes (LHCs), form PSI and PSII. Chl *a* is component of the RC and LHC complexes of both PSI and PII. The LHC comprises Chl *a*, Chl *b*, xanthophylls/carotenoid pigments, and proteins (Lhca1–Lhca4 in PSI, Lhcb1–Lhcb6 in PSII) which collectively form the antenna complex. Conversion of Chl *a* to Chl *b* is by two-step oxidation catalyzed by the enzyme chlorophyll *a* oxygenase (Cao) in rice (Morita *et al.*, 2005). Chl *b* is then converted back to Chl *a* by two-step reduction, thus constituting a cycle. The first reduction is by Chl *b* reductase which requires heterodimerization of non-yellow coloring 1 (*Nyc1*) and *Nyc1*-like (*Nol*) gene products in rice (Kusaba *et al.*, 2007; Sato *et al.*, 2009) to yield HmChl, and a second reduction is by HmChl reductase (Hcar), resulting in Chl *a* (Sakuraba *et al.*, 2013). The Chl cycle plays a crucial role in the greening process, light acclimatization, and leaf senescence.

Leaf senescence is the endogenously regulated degradation process resulting in irreversible yellowing and finally death of the organ. Several genes that were found to be highly differentially expressed during senescence have been referred to as senescence-associated genes (SAGs), and many of these have been used as molecular markers indicative of the process. Genetic variants of plants showing retention of leaf greenness and delay of senescence exhibit the stay-green phenotype (Hörtensteiner, 2006). Degradation of the photosynthetic pigment Chl *a* is one of the major events during leaf senescence. Stay-green mutants are ideal subject for studying Chl degradation (Cha *et al.*, 2002; Armstead, 2007; Jiang *et al.*, 2007; Morita *et al.*, 2009; Sato *et al.*, 2009; Schelbert *et al.*, 2009). Five categories of stay-green traits have been described (Thomas and Howarth, 2000), of which two categories, Type A and B, are of agronomic importance because the delay of senescence in Type A plants is due to delay in initiation whereas in Type B plants it is due to the slower progression of Chl degradation and loss of photosynthetic efficiency.

Interplay of phytohormones can regulate senescence, and it is known that cytokinins (CKs), auxins, and gibberellins (GA_3_) delay, while salicylic acid, jasmonic acid, abscisic acid (ABA), and ethylene accelerate leaf senescence. The auxin indole-3-acetic acid (IAA) is known to be involved in retarding senescence in detached senescing leaves of Arabidopsis (Noh and Amasino, 1999; Cohen *et al.*, 2003). In plants, IAA is synthesized by two major pathways, namely a tryptophan-independent pathway where indole-3-glycerol phosphate (IGP) is the direct precursor, and a tryptophan-dependent pathway where tryptophan derived from IGP is the precursor (Mano and Nemoto, 2012). In contrast, ethylene and ABA promote senescence. Exogenous application of ethylene promotes visible yellowing of leaves, and several ethylene biosynthesis genes are up-regulated during senescence (van der Graaf *et al.*, 2006). Ethylene is synthesized in a few highly regulated steps by conversion of *S*-adenosylmethionine to 1-aminocyclopropane-1-carboxylic acid (ACC) by ACC synthase, which is then converted to ethylene by ACC oxidase (ACO). Transgenic broccoli containing an antisense ACC oxidase gene showed major reduction in ethylene production and improvement in head color changes after harvest (Henzi *et al.*, 2000). ABA is converted to phaseic acid as a result of hydroxylation by ABA-8' hydroxylase (Ahs), a cytochrome P450 monoxygenase. The synthesis as well as the breakdown of ABA regulates its endogenous levels. A lesion-mimic rice mutant (*spl3*) is insensitive to ABA and shows delayed dark-induced and natural senescence (Wang *et al.*, 2015).

The plant hormone CK retards senescence in detached leaves and promotes greening in plants (Richmond and Lang, 1957; Kakimoto, 2003). Heterologous expression of the *Agrobacterium tumefaciens* isopentyl transferase (IPT) gene coding for an enzyme which catalyzes the rate-limiting step of CK production results in increased levels of endogenous CK in plants (Akiyoshi *et al.*, 1984). Transgenic plants of important crop species including rice, cassava, and cotton expressing the IPT gene under the *SAG12* promoter convincingly show that CK delays leaf senescence (Lin *et al.*, 2002; Zhang *et al.*, 2010; Liu *et al.*, 2012). The stay-green phenotype caused by overexpression of the IPT gene induced extreme drought tolerance (Rivero *et al.*, 2007) and delayed stem and leaf senescence (Ma and Liu, 2009). CKs play a role in preserving the structure and function of the photosynthetic machinery under stress conditions (Cherniad’ev, 2009). CK increases sink activities by stimulating assimilate accumulation in chloroplasts of older leaves (Criado *et al.*, 2009). The role of CK in the biosynthesis of Chl, stimulation of tetrapyrole biosynthesis, chloroplast transcription (Zubo *et al.*, 2008), and enhancement of photosynthetic efficiency has also been demonstrated (Yaronskaya *et al.*, 2006). CK has been reported to retard Chl degradation (Kao, 1980; Jordi *et al.*, 2000). However, the actual step of CK involvement in the Chl metabolic pathway and the mechanisms by which it could retard senescence are not known. An ethylmethane sulfonate (EMS)-induced, stay-green mutant (*N22-H-dgl162*) of the Nagina22 (N22) rice cultivar showing delayed senescence was earlier identified in a field screen under prolonged drought and heat conditions (Panigrahy *et al.*, 2011). The aim of this study was to investigate the mechanism of CK-mediated delay of dark-induced senescence (DIS) in rice, taking cues from the stay-green mutant. We studied the effect of 6-benzyl adenine (BA) on the photosynthetic apparatus in rice leaves during DIS by green gel analysis and HPLC profiling of the pigments and their identification by MS/MS analysis. We analyzed the transcriptome of BA-treated versus untreated N22 leaves to identify genes induced by CK during DIS. Real-time PCR analyses of genes associated with PSII, senescence, and the Chl cycle were carried out to understand the possible mechanism by which CK delays senescence in rice leaves.

## Materials and methods

### Plant growth conditions

For all experiments, rice seedlings were grown in similar size pots (five plants per pot) containing field soil, in a greenhouse with controlled temperature settings of 30 °C/25 °C during 16h light (20 µmol m^−2^ s^−1^ of photosynthetic photon flux)/8h dark cycle, respectively, and 60% constant humidity. Fifteen plants at the four-leaf stage were covered with cardboard boxes for dark treatment in the greenhouse. The middle portion of the third leaf from the apex was taken in all experiments. Treatment of detached leaves with a 2mg ml^−1^ solution of BA was as described by Jiang *et al.* (2007), followed by exposure to DIS at different time intervals ranging from 0h to 96h with or without BA. Dark treatment was used as a means to induce senescence.

### Photosynthesis measurement

Detached leaves were blotted dry after treatments at various time points and transferred into a leaf disc oxygen electrode chamber (LD-2; Hansatech Instruments Ltd, King’s Lynn, UK). The topmost capillary matting was moistened with 200 µl of 1M bicarbonate buffer (pH 9.0), which results in a gaseous atmosphere of ~5% (v/v) CO_2_ in the chamber. The leaf discs were arranged on this matting symmetrically in three successive rings of one, six, and 12. Oxygen in the chamber was calibrated for every sample as per the manufacturer’s instruction. Photosynthetic oxygen evolution was measured at 25 °C by a computer program, supplied by the manufacturer.

### Chlorophyll fluorescence measurement

A portable pulse amplitude-modulated fluorescence meter (Walz, Effeltrich, Germany) was used to obtain measurements of leaf Chl fluorescence. The measurements were taken at 20 °C and in green light. Initial (*F*_o_), maximal (*F*_m_), and variable (*F*_v_=*F*_m_–*F*_o_) fluorescence were determined directly after dark acclimation. To obtain *F*_m_, a light pulse of 2000 μmol m^−2^ s^−1^ was applied. The quantum yield under illumination [(*F*_m_'–*F*_t_)/*F*_m_'] was determined during the measurement. The experiment was carried out three times.

### Pigment extraction and analysis

Leaf samples (~200mg) were ground to a fine powder with liquid nitrogen, and pigments were extracted with 80% acetone. The samples were centrifuged at 15 000 *g* for 10min and the supernatant was used for Chl estimation and HPLC analysis (Roca *et al.*, 2004). Chls were determined according to Arnon (1949) using a Shimadzu UV-VIS spectrophotometer (model UV-2600, Japan). HPLC analysis of Chl pigments was done according to Jiang *et al.* (2007) by using an Agilent HPLC 1100 series equipped with a C-18 column (Waters Nova-Pak, 3.9×150mm) and a dual absorbance detector. Separation was carried out on an elution gradient with the mobile phases (A) ion pair reagent (1M ammonium acetate in water)/methanol (1:4, v/v) and (B) acetone/methanol (1:4, v/v), at a flow rate of 1.2ml min^−1^. The gradient was isocratic A for 4min, isocratic B for 20min, and a return to A for 6min, and detection was at 660nm. The absorption spectrum of each peak was obtained from their respective chromatograms in the HPLC profile. The eluted Chl pigment samples were collected in special eppendorf tubes. The HPLC-purified samples were vacuum centrifuged for 2h to a powdered form for further analysis.

### MS analysis

The dried pellet of HPLC-purified Chl pigments was dissolved in 50% acetonitrile (ACN) containing 0.1% trifluoroacetic acid (TFA), and 1 μl of matrix (5mg ml^–1^ 50% ACN containing 0.1% TFA) was spotted on the MALDI (matrix-assisted laser desorption ionization) target plate. The sample was allowed to air dry. The mass spectra were acquired using a 4800 MALDI TOF-TOF analyzer obtained from Applied Biosystems (Foster City, CA, USA). The mass spectrometer was ﬁtted with an Nd:YAG laser (355nm) to ionize samples at 200 Hz. The ion path lengths of linear, reﬂector, and MS/MS modes were 1.5, 3, and 2.4 m, respectively. The instrument consists of a high-energy collision-induced (CID) cell, and spectra were obtained using air as the CID gas with 1kV and 2kV energy in the positive ion mode.

### Microarray analysis

Total RNA was extracted with TRIzol reagent (Sigma Aldrich) from BA-treated and untreated detached N22 leaves after 72h of DIS and used for hybridization of a Rice Affymetrix gene-chip (51K arrays) containing probe sets designed from 48 564 japonica and 10 260 indica gene sequences according to the Affymetrix GeneChip expression analysis technical manual. Three biological replicates were used for the experiment. Annotation of the differentially expressed probes was done using NetAffyx software of Affymetrix and further validated using BLASTX search through NCBI. The microarray data have been submitted to the GEO repository; they were assigned the GEO accession number GSE55902, and can be viewed at http://www.ncbi.nlm.nih.gov/geo/query/acc.cgi?acc=GSE55902

### Quantitative PCR (qPCR) analysis

Total RNA from rice leaves was treated with RNase-free DNase I (Invitrogen) to remove DNA and used for reverse transcription with a superscript-III cDNA synthesis kit (Invitrogen). Real-time PCR was performed as described previously (Jisha *et al.*, 2015). Rice *OsActin1* was used as internal control, and relative gene expression levels were calculated using the 2^−ΔCT^ method [–ΔC_T_ indicates –(C_T_ of target–C_T_ of *OsActin1*), and C_T_ is the threshold cycle number of the amplified gene].

### Native gel electrophoresis of chlorophyll–protein complexes

The native Chl–protein complexes were separated as described by Allen and Staehelin (1991) with some modifications. To isolate thylakoid membranes, leaves were ground using a mortar and pestle with liquid nitrogen in an ice-cold grinding buffer (50mM HEPES, pH 7.6, 0.3M sorbitol, 10mM NaCl, 5mM MgCl_2_), filtered through two layers of Miracloth, and centrifuged at 3000 *g* for 5min at 4 ^o^C. The supernatant was centrifuged at 20 000 *g* for 7min and the pellet was then washed twice and resuspended in buffer containing 50mM HEPES, pH 7.6, 0.1M sorbitol, 10mM NaCl, 5mM MgCl_2_.

For electrophoresis, the resolving gel (8.0%) contained 25mM TRIS-HCl (pH 8.8) and 10% glycerol, while the stacking gel (4.0%) contained 25mM TRIS-HCl (pH 6.8) and 10% glycerol. The electrode buffer contained 25mM TRIS, 192mM glycine (pH 8.3), and 0.1% SDS. Before electrophoresis, the isolated thylakoid membrane suspension was centrifuged and the pellet was washed twice in 2mM TRIS-maleate (pH 7.0) (Oh *et al.*, 2003) and resuspended in solubilization buffer (4% digitonin, l% glycerol). The samples were kept on ice for 30min, and insoluble materials were removed by centrifugation at 15 000 *g* for 10min. The samples were equally loaded onto each well and then normalized to the fresh weight; following electrophoresis, the green gel was photographed under visible light.

### Western blot analysis

Rice leaves were ground into fine powder in liquid nitrogen and protein was extracted with 5ml of ice-cold grinding buffer (50mM HEPES, pH 7.6, 0.3M sorbitol, 10mM NaCl, 5mM MgCl_2_) per 150mg FW of leaves. The homogenate was filtered through two layers of Miracloth and centrifuged at 3000 *g* for 5min at 4 °C to remove the debris. The protein content was determined by Bradford assay (Biorad) and 50 µg of protein for each sample were diluted to 1× concentration using 5× SDS loading buffer (Takara) and subjected to SDS–PAGE after boiling. The proteins were separated by 10% SDS–PAGE, and electrotransferred to polyvinylidene fluoride (PVDF) membranes (Amersham) according to standard procedures. Blots were probed with rabbit anti-PsbP [a 23kDa protein from the oxygen-evolving complex (OEC) of PSII] antibodies (1:2000) and detection was with horseradish peroxidase (HRP)-conjugated goat anti-rabbit secondary antibodies (1:5000) followed by the ECL-Plus Western blotting Detection system (Roche) according to the manufacturer’s instructions. Antibodies were from Agrisera.

### Statistics

The data presented are the mean values (±SE) of results from 3–4 experiments conducted on different days. The data were subjected to further statistical significance by one-way ANOVA using SigmaPlot Version 11.0.

### Primers

Primers used in this study are listed in Supplementary Table S1 at *JXB* online.

## Results

### Cytokinin-mediated changes in physiological parameters during DIS in rice

**Fig. 1.**
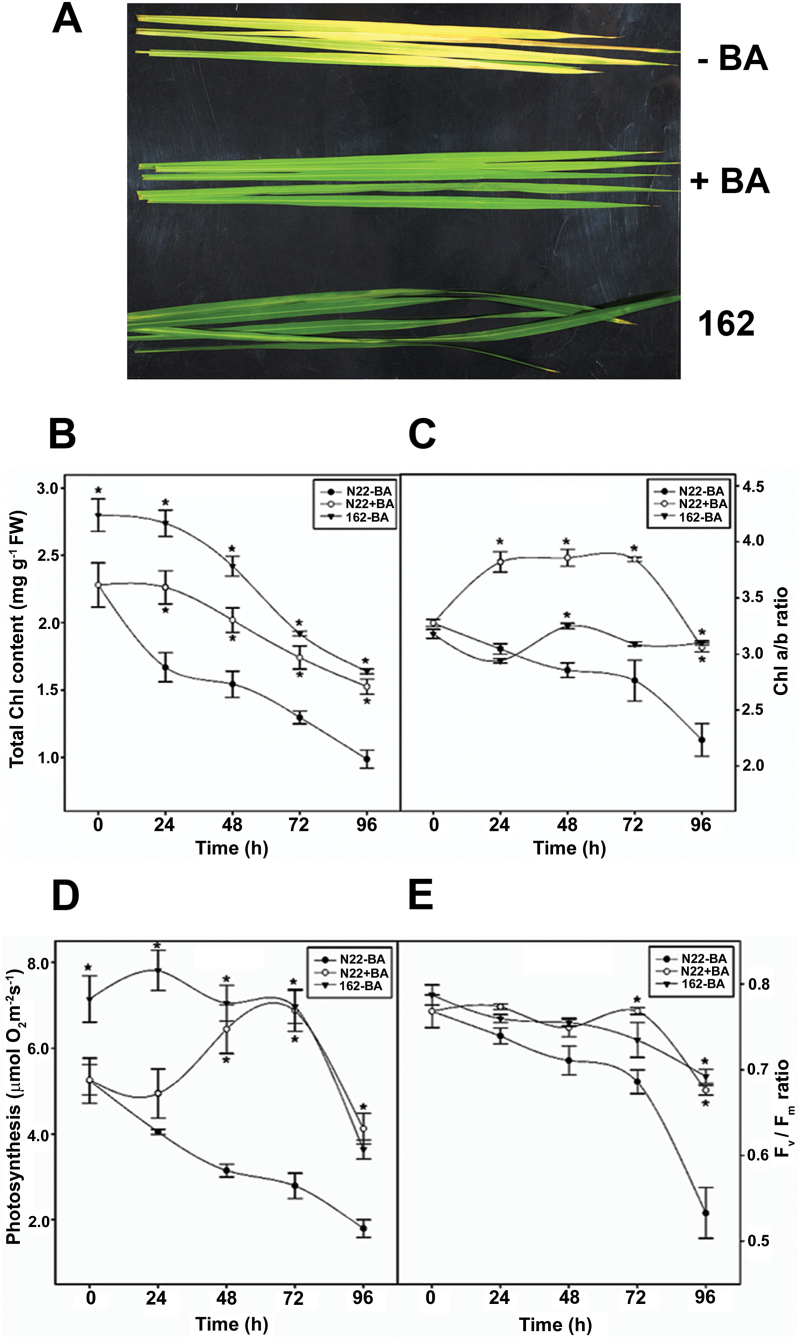
Effect of cytokinin treatment on rice leaves. (A) Phenotypic appearance of 4-week-old detached leaves of N22 with (+BA) or without BA (–BA) and *N22-H-dgl162* (162) incubated in the dark for 5 d. (B) Total chlorophyll (mg g^–1^ FW leaves). (C) Chl *a*/*b* ratio. (D) Photosynthetic oxygen evolution (µmol O_2_ m^−2^ s^−1^). (E) Chlorophyll fluorescence parameters expressed in terms of the *F*_v_/*F*_m_ ratio in detached N22 rice leaves with and without BA treatment and *N22-H-dgl162* leaves at different time intervals of dark incubation. Data represents mean values (± SE) from at least four independent experiments. Asterisks indicate statistically significant differences (*P*<0.05) between the control and BA-treated N22/*N22-H-dgl162* cut leaves. (This figure is available in colour at *JXB* online.)

Detached leaves of an EMS-induced, stay-green mutant (*N22-H-dgl162*) of the drought- and heat-tolerant rice cultivar N22 remained green, whereas detached leaves of N22 turned yellow when subjected to DIS for 5 d (Fig. 1A). Exogenous application of CK to detached leaves has been shown to prevent senescence (Richmond and Lang, 1957). Treatment of detached N22 leaves with BA, a synthetic CK, retarded yellowing, and the leaves remained green even after 120h of DIS (Fig. 1A). Thus, treatment of N22 leaves with BA maintained greenness and retarded yellowing during DIS.

During the ontogenic progress of senescence, there is a sequential loss of Chl content and photosynthetic functions in barley leaves (Wiedemuth *et al.*, 2005). The total Chl level was higher at all time points in *N22-H-dgl162* and BA-treated N22 leaves when compared with untreated N22 leaves (Fig. 1B). The Chl *a/b* ratio increased from 3.0 to 4.0 in BA-treated leaves after 24h of DIS and remained constant till 72h, followed by a slight decrease at 96h. In the untreated N22 leaves, the Chl *a/b* ratio decreased from 3.0 to 2.2 after 96h of DIS. In *N22-H-dgl162* leaves, the ratio remained constant at ~3.0 even after 96h of DIS (Fig. 1C). Thus, maintaining both a higher Chl level and a higher Chl *a/b* ratio distinguishes the mutant and BA-treated N22 leaves from untreated senescing N22 leaves.

The functionality of stay-greenness in mutant and BA-treated N22 leaves was evaluated during DIS by measuring the rates of photosynthetic oxygen evolution and characteristics of Chl fluorescence (*F*_v_/*F*_m_)_._ Photosynthetic oxygen evolution measurements showed that the rate decreased from 5.4 µmol O_2_ m^−2^ s^−1^ at 0h to 3.6 µmol O_2_ m^−2^ s^−1^ in BA-treated N22 and from 7.0 µmol O_2_ m^−2^ s^−1^ to 4.5 µmol O_2_ m^−2^ s^−1^ in *N22-H-dgl162* leaves after 96h of DIS. In the untreated N22 leaves, the rate of O_2_ evolution decreased steadily to 2 µmol O_2_ m^−2^ s^−1^ after 96h of DIS (Fig. 1D). The *F*_v_/*F*_m_ ratio is indicative of maximum efficiency of the PSII photochemical process. The *F*_v_/*F*_m_ ratio remained constant at ~0.7 in *N22-H-dgl162* and BA-treated N22 leaves but decreased steadily to 0.5 in untreated N22 leaves after 96h of DIS (Fig. 1E). This result indicates that after 96h of DIS the PSII apparatus functioned more efficiently in *N22-H-dgl162* and BA-treated N22 leaves than in untreated N22 leaves.

### Enhanced greenness under dark conditions in CK-treated leaves of rice is accompanied by accumulation of chlorophyll intermediates

**Fig. 2.**
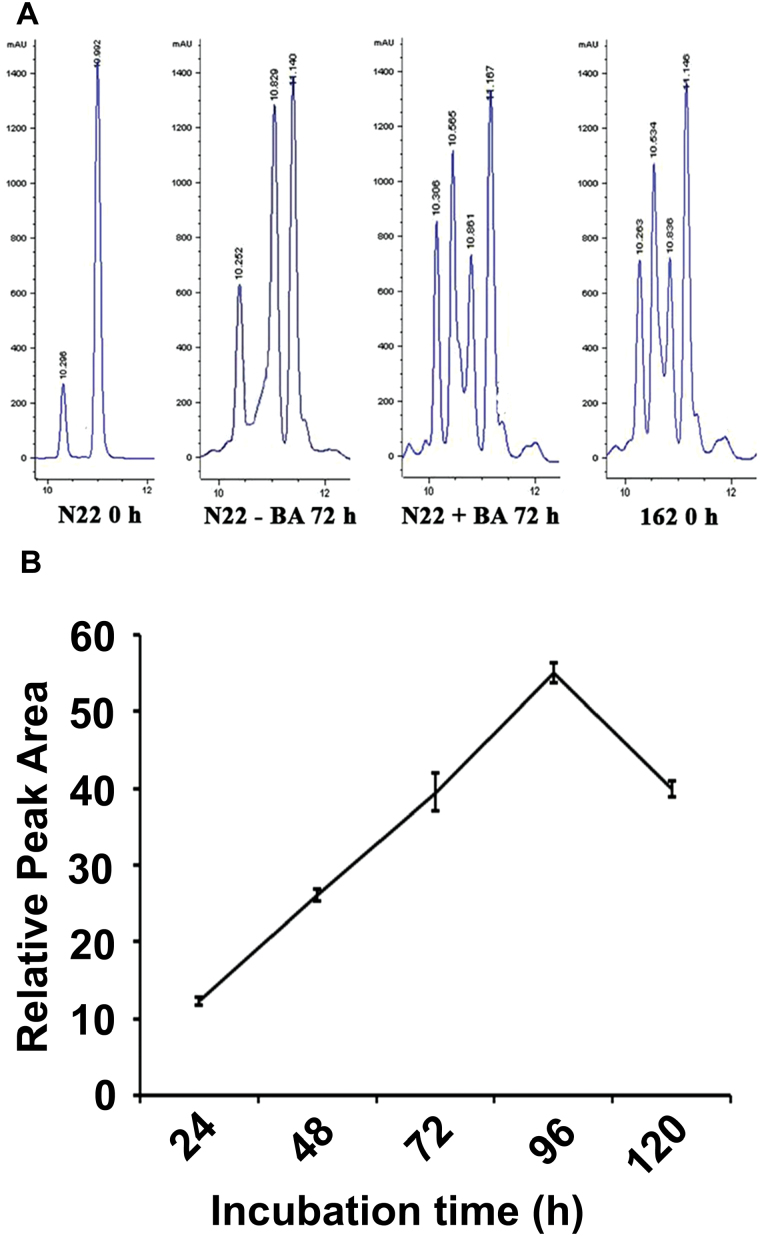
Photosynthetic pigment profiles of rice leaves. (A) HPLC profile of chlorophyll pigments from leaves of 4-week-old rice plants. Total chlorophyll was extracted from cut leaves of N22 before (N22 0h) and after dark incubation for 72h with (N22 +BA) or without (N22 –BA) BA treatment and untreated *N22-H-dgl162* (162 0h). Peaks are numbered according to their retention time in minutes. Similar results were reproduced in at least five independent experiments. (B) Kinetics of relative peak area of the additional peak at ~10.5min observed in N22 +BA and *N22-H-dgl162* HPLC profiles following 0–120h of dark treatment. Similar results were reproduced in at least three independent experiments.

The photosynthetic pigment profiles were analyzed by HPLC in order to study the stability of Chl pigments. Pigments extracted from fresh N22 leaves (0h control) showed two peaks at 660nm in the HPLC chromatogram, which correspond to Chl *b* and Chl *a* based on the absorption spectra, with retention time (*t*_ret_ min^–1^) values of 10.3 and 10.9, respectively (Fig. 2A). In addition to the two peaks obtained in the 0h control, the leaves without BA treatment showed a peak at 11.1 whereas BA-treated leaves and *N22-H-dgl162* leaves showed two peaks at 10.5 and 11.1 after 72h of DIS (Fig. 2A). To study the kinetics of the 10.5 peak accumulation, peak area was calculated from HPLC runs of BA-treated N22 samples at different time intervals of DIS. The accumulation kinetics showed a linear increase in the 10.5 peak area from 24h to 96h, with a decline only at 120h of dark incubation (Fig. 2B). It is interesting to note that it is during this 96–120h period that leaves begin to turn yellow.

**Fig. 3.**
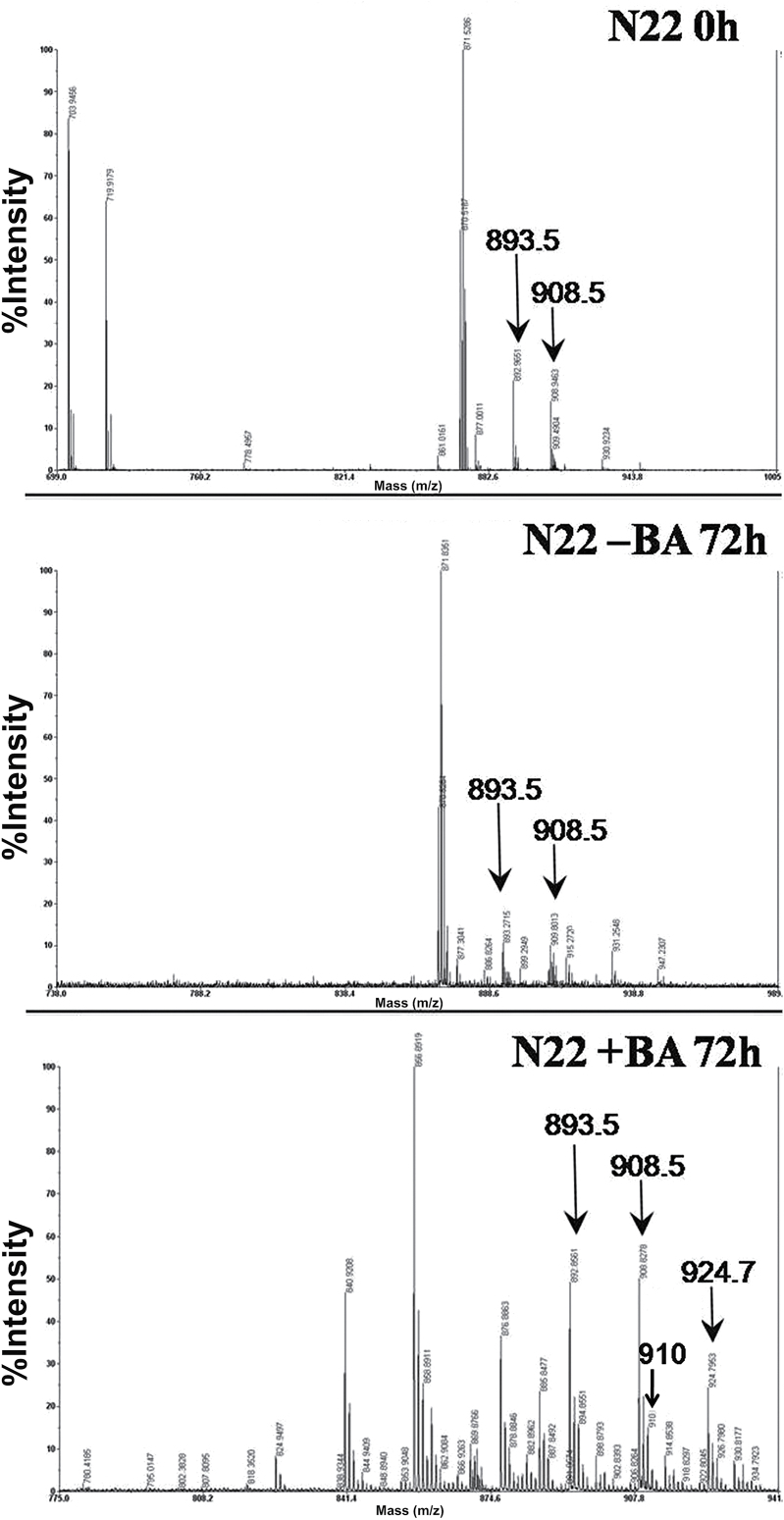
Mass spectra of HPLC-fractionated samples from N22 leaves at 0h and 72h of DIS without and with BA treatment. Three independent experiments were performed and only the consistently occurring peaks are labeled.

MS was used to identify the Chl derivatives obtained by HPLC fractionation. One or more ionic species in solution can be detected and characterized based on the *m/z* values obtained by electron spray ionization (ESI)-MS. The pigment fractions separated by HPLC for individual samples were pooled and analyzed by applying positive ion mode. N22 leaves at 0h showed two ionic species, Chl *b* (908.5) and Chl *a* (893.5), corresponding to the two HPLC peaks at 10.3 and 10.9 *t*_ret_ min^–1^, respectively (Fig. 3). N22 leaves without BA treatment after 72h of DIS showed three ionic species with *m/z* values of 893.5, 908.5, and 915 corresponding to Chl *b*, Chl *a*, and the sodium adduct of Chl *a*, respectively. On the other hand, BA-treated N22 leaves after 72h of DIS showed several additional peaks, the most consistent being ionic species with *m/z* values of 910 and 925, which correspond to 7-hydroxymethyl Chl *a* (910) and hydroperoxy Chl *a* (925).

**Fig. 4.**
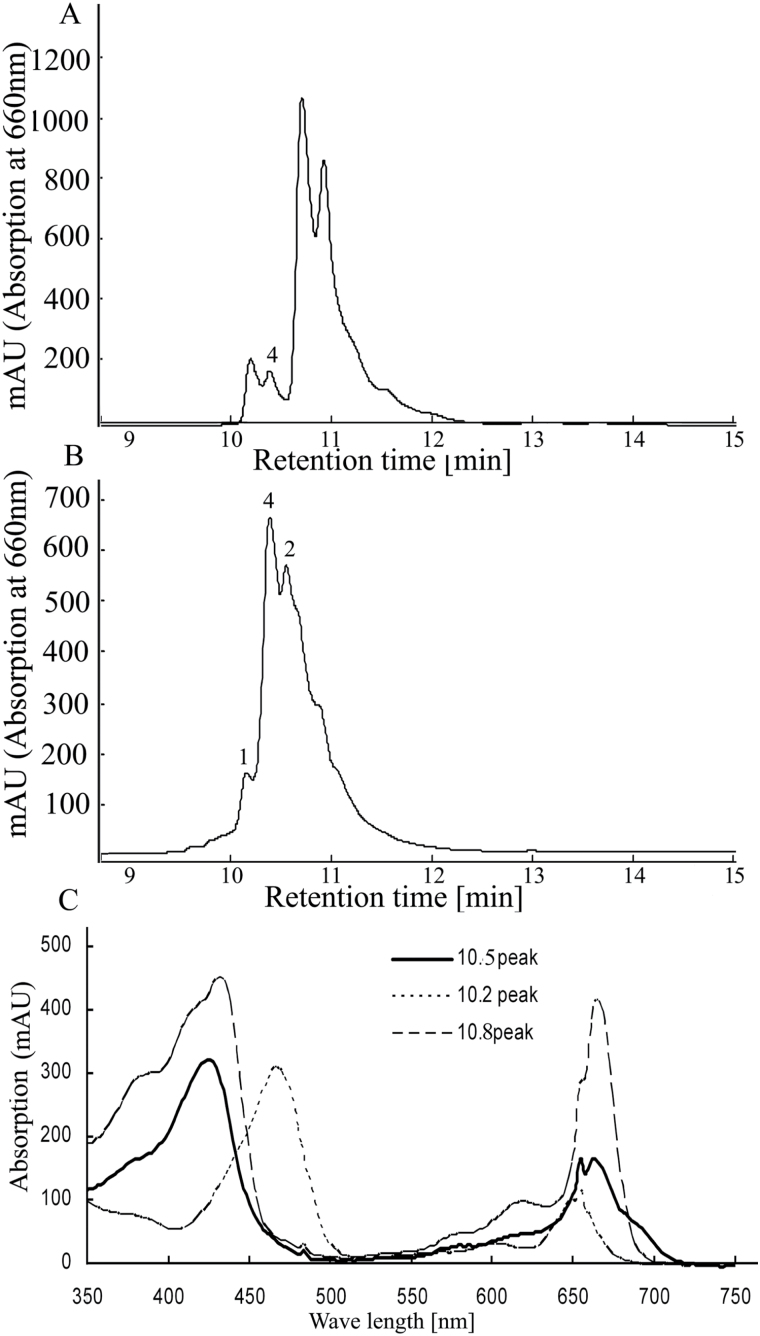
HPLC profile of the authentic 7-hydroxymethyl chlorophyll prepared from leaf chlorophyll extract of N22 in (A) 8-hydroxyquinoline (8-HQ) and methanol; and (B) 8-HQ, methanol, and NaBH_4_. (C) Absorption spectra of the peaks with retention times 10.2, 10.8, and 10.5min observed in the HPLC profiles.

It is known that treatment of Chl *b* with sodium borohydride (NaBH_4_) results in the formation of HmChl (Ito *et al.*, 1996). In order to confirm the identity of the accumulated intermediate, authentic HmChl was prepared by reduction of Chl *b* with NaBH_4_ and 8-hydroxyquinoline (8-HQ) in methanol. N22 leaves treated with 8-HQ and methanol alone showed a minor peak at 10.5 *t*_ret_ min^–1^ (Fig. 4A), which can be explained by the fact that addition of 8-HQ stabilizes HmChl. Addition of NaBH_4_ resulted in a major peak at retention time 10.5min and two other peaks at 10.2min and 10.8min (Fig. 4B). The absorption spectrum of the 10.5min peak in BA-treated samples matched that of HmChl (Ito *et al.*, 1996; Fig. 4C). These results confirmed that HmChl accumulated in the dark-induced Chl extracts of BA-treated samples of N22 at 72h of DIS.

### Cytokinin-mediated molecular events in delay of DIS

To gain insight into the molecular events in the delaying of DIS by CK, microarray analysis of BA-treated versus untreated N22 control samples was carried out using the Affymetrix 3'UTR (untranslated region) rice genome array consisting of 57 381 probe sets. Genes showing a >1.5-fold difference with a *P*-value <0.05 from three biological replicates were selected. Up-regulated pathways included CK regulation, auxin biosynthesis, nitrogen metabolism, and response to oxidative stress, whereas cellulose biosynthetic, carbohydrate metabolic, ethylene-responsive, and GABA (γ-aminobutyric acid) shunt pathways were down-regulated. The details of differentially regulated genes are listed in Table 1.

**Table 1.** Differential expression of genes involved in various metabolic pathways upon treatment with cytokinin during DIS

Broad functional category	Gene locus ID	Gene name	Fold change
Cytokinin metabolism	LOC_Os01g10110	CK dehydrogenase/oxidase	19.6
	LOC_Os01g72330	OsRR4 type-A response regulator	3.0
	LOC_Os04g36070	OsRR1	3.1
	LOC_Os03g08624	Dihydroflavonol-4-reductase	2.4
	LOC_Os01g13610	Isoflavone reductase	2.9
ABA metabolism	LOC_Os08g36860	ABA hydroxylase	7.0
	LOC_Os12g17880	Ubiquitin protein ligase	2.58
Ethylene metabolism	LOC_Os11g37520	Ethylene overproducer 1-like	–1.9
	LOC_Os02g53180	ACC oxidase	-4.0
	LOC_Os06g03990	ACC synthase	-1.7
Auxin metabolism	LOC_Os03g03450	Anthranilate phosphoribosyltransferase	2.2
	LOC_Os09g08130	Indole-3-glycerol phosphate synthase	8.1
	LOC_Os07g08430	Tryptophan synthase alpha-subunit	2.4
	LOC_Os06g42560	Tryptophan synthase beta chain 2	1.8
	LOC_Os02g42350	Indole-3-acetonitrile nitrilase	1.7
	LOC_Os01g06660	Indole-3-pyruvic acid decarboxylase	3.5
	LOC_Os12g32750	Tryptamine monooxygenase	–2.0
PSII complex	LOC_Os07g04840	PSBP-1, 23kDa oxygen evolving complex	2.1
	LOC_Os07g37030	Cytochrome b6-f complex iron–sulphur subunit	2.2
	LOC_Os07g37240	LHCB4.2 chlorophyll A-B binding protein	2.6
	LOC_Os01g31690	PSBO1, oxygen-evolving enhancer protein 1	2.3
	LOC_Os04g38410	Lhcb6 chlorophyll A-B binding protein	3.2
	LOC_Os12g19470	Ribulose bisphosphate carboxylase small chain	2.9
	LOC_Os12g19381	Ribulose bisphosphate carboxylase small chain	2.5
GABA metabolism	LOC_Os08g36320	Glutamate decarboxylase	–2.6
	LOC_Os03g44150	Aminotransferase	–1.6
Carbohydrate metabolism	LOC_Os02g34560	Plant neutral invertase	2.6
	LOC_Os11g07440	Plant neutral invertase	3.0
	LOC_Os01g53930	Hexokinase	–1.6
	LOC_Os11g31190	Nodulin MtN3 family protein	–3.6
	LOC_Os12g29220	SAG29	–14.5
	LOC_Os03g11420	beta-Glucosidase (SAG)	–3.9
Chlorophyll metabolism	LOC_Os04g41260	Protoporphyrinogen oxidase	–2.9
Nitrogen metabolism	LOC_Os04g45970	Glutamate dehydrogenase (NAD+)	2.8
	LOC_Os07g46460	Glutamate synthase	2.2
Oxidative stress	LOC_Os10g38340	Glutathione *S*-transferase	4.2
	LOC_Os01g72140	Glutathione *S*-transferase	10.5
	LOC_Os01g27390	Glutathione *S*-transferase	9.9
	LOC_Os10g38600	Glutathione *S*-transferase	5.7
	LOC_Os10g38360	Glutathione transferase	5.1
	LOC_Os10g38640	Glutathione *S*-transferase 4	4.2
	LOC_Os10g38340	Glutathione transferase	4.2
	LOC_Os10g38780	Glutathione *S*-transferase	2.8
	LOC_Os01g27210	Glutathione *S*-transferase	2.1
	LOC_Os04g17050	Glutaredoxin/GST	2.1
	LOC_Os09g23370	Glutathione-dependent-formaldehyde	1.9
	LOC_Os01g72160	activating enzyme	1.9
	LOC_Os12g32650	Glutathione transporter	1.8
Triacylglycerol degradation	LOC_Os01g46370	Triacylglycerol lipase	–1.9
	LOC_Os05g11910	GDSL-like lipase/acylhydrolase	–8.4
	LOC_Os06g06290	GDSL-like lipase/acylhydrolase	–9.5
Transport genes	LOC_Os02g17500	Sugar transporter	–1.9
	LOC_Os05g04610	ABC transporter	–2.6
	LOC_Os01g70490	Potassium transporter	–1.6

Two genes of the A-type two-component CK signal transduction system (*OsRR1* and *OsRR4*) and CK-responsive genes such as cytokinin dehydrogenase/oxidase (*Cdg*), isoflavanol reductase, and dihydroflavanol reductase were up-regulated in the BA-treated N22 leaves, suggesting a negative feedback regulation commonly observed in many cell signaling systems (Hirose *et al.*, 2007; Tsai *et al.*, 2012) and homeostatic control of CK activity (Wang *et al.*, 2011).

Several genes related to the biosynthesis of the auxin IAA via different pathways were up-regulated in BA-treated leaves during DIS (Table 1). The levels of ABA-8' hydroxylase and a ubiquitin-ligase gene negatively regulating ABA biosynthesis were increased 7-fold and 2.5-fold, respectively, suggesting increased ABA catabolism upon BA treatment. The ethylene biosynthetic genes, ACC synthase and ACC oxidase, were down-regulated in BA-treated rice leaves under DIS. This indicates that the auxin pathway is up-regulated while ABA and ethylene pathways are down-regulated by CK during DIS.

Microarray analysis showed higher expression of several genes associated with PSII of the photosynthesis light reaction in BA-treated leaves when compared with untreated N22 leaves during DIS. These included *Lhcb4* and *Lchb6*, genes encoding Chl *a/b*-binding proteins (*Cbp*) from the LHC, and the oxygen-evolving enhancer genes *PsbO* and *PsbP* from the OEC of PSII. In addition, the cytochrome *b*_6_-*f* complex subunit, a component of the light reaction of photosynthesis, was up-regulated. Thus, CK regulates the light-harvesting and oxygen-evolving functions in the chloroplast. However, protoporphyrinogen oxidase (*Ppo*) involved in Chl biosynthesis showed lower expression. We also found that expression of Rubisco genes, glutamate synthase, and glutamate dehydrogenase genes involved in nitrogen metabolism were increased upon treatment of N22 leaves with BA during senescence induced by dark treatment. The apparent increased expression of photosynthesis-related genes in BA-treated leaves may well be due to a decrease in the transcripts in the untreated control. It has been shown previously that a high yield of the rice cultivar Akenohoshi was due to maintenance of *rbcL* and *rbcS* transcript levels during senescence, whereas the lesser yielding Nipponbare showed a decline in these transcripts and CK could account for the difference in reduction of Rubisco during senescence between cultivars (Ookawa *et al.*, 2004).

 SAGs which have been reported to be up-regulated during senescence in earlier studies were found to be down-regulated in BA-treated leaves under DIS. These include glutamate decarboxylase (*Gad*) and aminotransferase (*Amt*) involved in the GABA shunt pathway (Ansari and Chen, 2009), SAG 29, the SWEET gene (*Mtn*), hexokinase (*Hks*), and β-glucosidase (*Bgs*) involved in sugar metabolism, and a group of genes encoding nutrient transporters such as a sugar transporter gene, ABC transporter, and potassium transporter. Our results showed that among the SAGs known to be down-regulated during senescence but found to be up-regulated upon BA treatment were two invertases (*Inv*) involved in sugar metabolism. Increased expression of invertase has been reported to be required for delay of senescence mediated by CK in tobacco (Lara *et al.*, 2004).

**Fig. 5.**
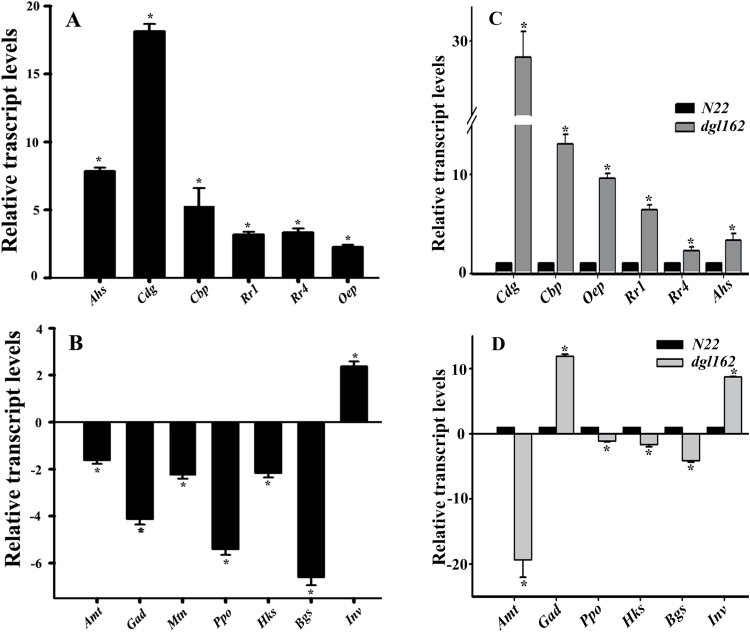
Transcript levels of genes differentially expressed in microarray analysis. Total RNA was extracted from N22 leaves subjected to 72h of DIS, with and without BA treatment (A, B) or the third youngest leaf of 48-day-old N22 and *N22-H-dgl162* rice plants (C, D). Real-time PCR was performed with primers for genes which include (A, C) phytohormone- and PSII-related: ABA hydroxylase (*Abs*), CK dehydrogenase (*Cdg*), OsRR1 (*Rr1*), OsRR4 (*Rr4*), Lhcb4 chlorophyll A-B binding protein (*Cbp*), and PSBP-1, 23kDa oxygen-evolving complex (*Oep*); (B, D) senescence-associated genes (SAGs): aminotransferase (*Amt*), glutamate decarboxylase (*Gad*), SAG29 (*Mtn*), protoporphyrinogen oxidase (*Ppo*), hexokinase (*Hks*), β-glucosidase (*Bgs*), and plant neutral invertase (*Inv*). *Actin* was used as internal standard. Data represent the mean ±SE from three independent biological replicates, and an asterisk indicates a *P*-value of 0.05.

We analyzed the expression of genes representative of various functions by qPCR. The expression of *Cdg*, *OsRR1*, and *OsRR4*, genes involved in CK metabolism and signaling, and *Ahs* involved in ABA degradation was higher and PSII-related genes such as *Cbp* (*Lhcb4*) and *Oep* also showed higher expression in BA-treated samples during 72h DIS (Fig. 5A). On the other hand, *Amt*, *Gad*, *Mtn*, *Hks*, and *Bgs* genes involved in sucrose metabolism and *Ppo* involved in Chl biosynthesis showed reduced expression, while the *Inv* gene which is normally down-regulated during senescence showed increased expression in BA-treated leaves following 72h of DIS (Fig. 5B). We also compared the expression of these genes during natural leaf senescence. Similar results were obtained when the expression of genes from naturally senescing third youngest N22 leaves from 48-day-old plants were compared with still green *N22-H-dgl162* leaves of the same age and position in the plant (Fig. 5C, D), suggesting similarity of events during natural senescence and DIS. The only exception was expression of the *Gad* gene in *N22-H-dgl162* leaves, indicating that glutamate decarboxylase which converts glutamate to GABA is up-regulated only during natural senescence (Fig. 5D). It has been reported that GABA may have a role as a signal molecule in co-ordinating carbon:nitrogen balance during developmental and not dark- or starvation-induced senescence (Buchanan-Wollaston *et al.*, 2005).

### Expression levels of genes involved in Chl *a*/*b* conversion

**Fig. 6.**
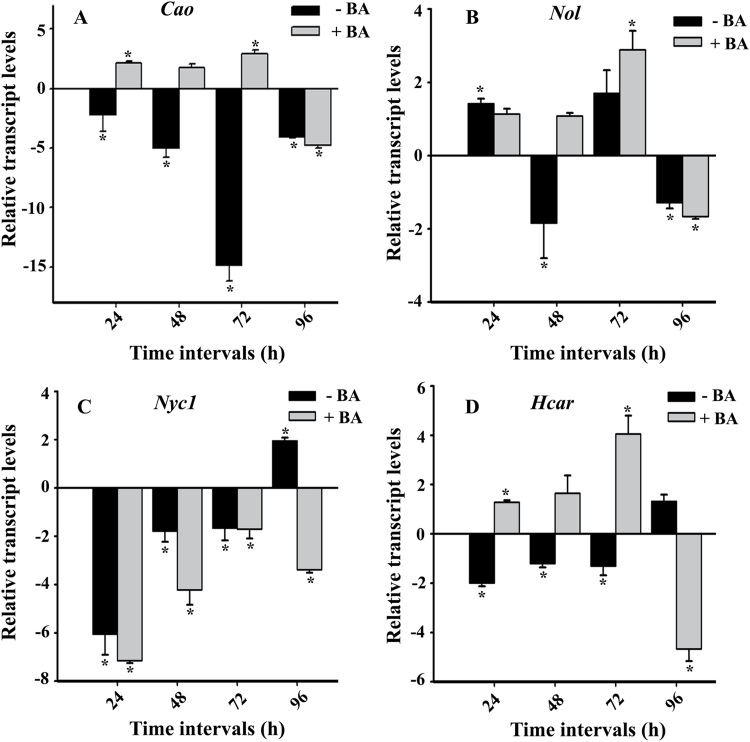
Expression pattern of genes encoding enzymes involved in the Chl *a*/*b* cycle. Total RNA was extracted from N22 leaves without and with BA treatment for 0, 24, 48, and 72h of dark incubation. Real-time PCR was performed with primers for (A) *Cao* (chlorophyllase *a* oxygenase); (B) *Nol* (*Nyc1*-like); (C) *Nyc1* (non-yellow coloring 1); and (D) *Hcar* (hydroxymethyl *Chla* reductase). Primer details are given in Supplelemtary Table S1. *OsActin* was used as control.

Expression levels of the four genes involved in the Chl *a/b* cycle were analyzed by qPCR to follow the kinetics of regulation. BA-treated N22 leaves were compared with untreated N22 leaves at 0, 24, 48, 72, and 96h of DIS. Expression of the *Actin* gene was used as an internal control to normalize the expression levels in all the samples. There was an increase in expression of *Cao* within 24h of BA treatment, with the level remaining high till 72h of DIS, whereas the level decreased in untreated samples after 72h of DIS when compared with untreated non-DIS control, suggesting that *CaO* is repressed under senescence. *Nol* and *Hcar* showed a similar pattern of expression to *CaO*, being strongly expressed 72h after DIS in response to BA treatment, and showed a decrease in expression in DIS leaves compared with untreated, non-DIS control (Fig. 6). *Nyc1* showed reduced expression in both untreated and BA-treated samples, indicating that it is strongly repressed during dark treatment.

### Cytokinin stabilizes chlorophyll–pigment complexes

**Fig. 7.**
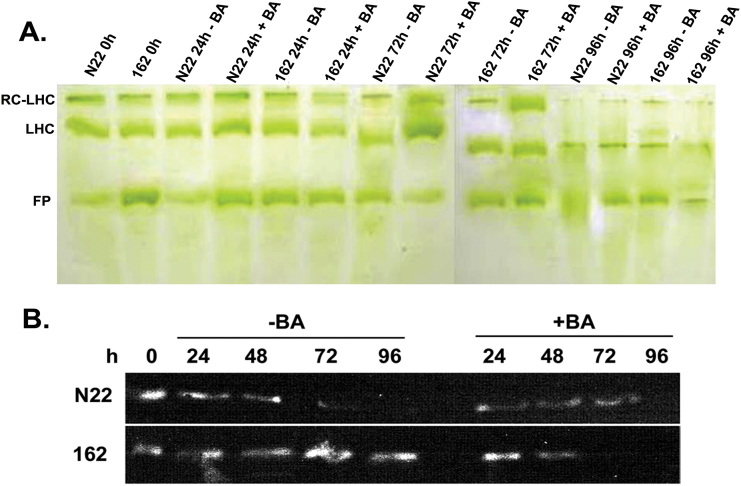
Electrophoretic analysis of pigment–protein complexes. (A) Green gel analysis of thylakoid membranes isolated from cut leaves of N22 and *N22-H-dgl162* incubated in the dark with or without BA treatment at different time intervals and solubilized with a detergent. Samples loaded into each well of the non-denaturing native gel were normalized to the fresh weight of leaves. Labels indicate: RC-LHC, reaction center–light harvesting complex; LHC, light-harvesting complex; FP, free pigment. (B) Western blot analysis of PsbP protein from untreated and BA-treated leaves of N22 and *N22-H-dgl162* leaves at 0h and different times of dark incubation. Extracts were prepared in 5ml of extraction buffer for 150 µg FW of leaf for each sample and 50 µg of protein was loaded on each lane after estimation using the Bradford method. Anti-Psb antiserum was used to detect protein, and a single band of ~23kDa was obtained. (This figure is available in colour at *JXB* online.)

Microarray analysis indicated that PSII-related genes were differentially expressed, and Chl analysis revealed that Chl *a/b* ratios and *F*_v_/*F*_m_ values were maintained during DIS in *N22-H-dgl162* and BA-treated N22 leaves, suggesting intactness of pigment–protein complexes. To study the effect of BA treatment on the stability of Chl*–*protein complexes, we performed non-denaturing green gel analysis. In the untreated leaves at 0h, three distinct major bands representing the RC–LHC complex, the LHCs, and free pigments were observed (Fig. 7A). In the untreated N22 leaves, the complexes were drastically reduced by 72h of DIS and degraded by 96h of DIS, whereas all the bands remained intact in *N22-H-dgl162* and BA-treated N22 leaves after 96h of DIS (Fig. 7A). However, BA treatment of *N22-H-dgl162* affected the complexes, and degradation was observed at 96h of DIS. Hence, Chl–pigment complexes were stable in *N22-H-dgl162* and BA-treated N22 leaves during DIS in rice leaves.

Western blot analysis was performed to examine the change in the amount of PsbP, a component of the OEC of PSII which showed a 5-fold increase in transcript levels in microarray analysis. In N22, the PsbP levels remained constant until 72h following BA treatment during DIS, by which time degradation was observed in the untreated samples (Fig. 7B). The level of Psb protein was maintained in the *N22-H-dgl162* mutant without BA treatment even at 96h of DIS, whereas BA treatment caused degradation by 72h of DIS. These results were in agreement with the degradation of pigment–protein complexes observed using green gel analysis. The changes in protein level support the possibility that the increase in transcript levels of photosynthetic genes in BA-treated N22 samples in comparison with untreated controls could be because of the degradation of transcripts in untreated controls rather than up-regulation of the genes.

## Discussion

The plant hormone CK is known to delay senescence in plants. BA-treated detached N22 leaves and untreated detached leaves of the *N22-H-dgl162* mutant showed a delay in senescence when subjected to DIS, a commonly used procedure for artificially inducing senescence by dark treatment of detached leaves to obtain uniformity and save time while performing experiments (Oh *et al.*, 2003; Chrost *et al.*, 2004). The physiological parameters indicated that delays in initiation of Chl degradation and loss of photosynthetic efficiency contribute to delayed senescence. This signifies the Type A category of stay-greenness in *N22-H-dgl162*, an agronomically important trait for crop productivity (Hörtensteiner, 2009). Further characterization of *N22-H-dgl162* would help to identify the mutation.

Our microarray data showed a higher level of transcripts for several plastid-localized photosynthesis-related genes such as Rubisco activase, Rubisco small chain, cytochrome *b*_6_-*f* complex, *PsbO*, *PsbP*, and *Cpb* in BA-treated leaves when compared with untreated leaves during DIS. Also the level of PsbP protein remained constant upon BA treatment during DIS. On the other hand, a Chl biosynthetic gene (*Ppo*) was down-regulated, suggesting that BA treatment maintains Chl levels by preventing degradation rather than inducing its synthesis, and protects the photosynthetic machinery during DIS, similar to the effect reported in wheat (Zavaleta-Mancera *et al.*, 2007).

Porra *et al.* (1994) showed by MS and ^18^O labeling that an oxygenase as opposed to a hydratase mechanism is operational in a two-step conversion of Chl *a* to Chl *b*. HPLC and MS/MS data revealed additional peaks in BA-treated leaves with *m/z* values of 910 and 925, suggesting accumulation of HmChl and aldehyde hydrate derivatives of Chl *a* during DIS. *CaO*, coding for the enzyme which catalyzes the oxidation of Chl *a* to Chl *b* in the two-step process, is up-regulated following BA treatment within 24h of DIS, suggesting that CK enhances the conversion of Chl *a* to Chl *b.* The appearance of an additional peak in HPLC and accumulation of HmChl and aldehyde hydrate species of Chl *a* in the leaf tissues also occurs at the same time. Also, we found that transcript levels of *Hcar* and *Nol* were reduced during DIS in untreated N22 samples, whereas BA-treated samples showed increased expression of both genes from 24h, with maximum expression at 72h, suggesting their role in Chl turnover. *Hcar* and *Nol* are reported to be drastically down-regulated during DIS and highly up-regulated during greening of etiolated seedlings (Sakuraba *et al.*, 2013). Overexpression of *Cao* (Tanaka *et al.*, 2001), as well as a decrease in Chl *a/b* ratios under low light intensities (Tanaka and Tanaka, 2011), has been reported to affect enlargement of the antenna size of PSII. Although BA treatment resulted in higher levels of *Cao*, this did not affect the Chl *a/b* ratios, suggesting maintenance of antenna size during DIS. The increased expression of *Cao* did not affect the Chl *b* levels during DIS, probably because of the feedback mechanism mediated by its N-terminal domain (Yamasato *et al.*, 2005) and also due to increased levels of other enzymes in the Chl cycle. Green gel analysis showed intact pigment–protein complexes in BA-treated leaves in comparison with untreated leaves during DIS. All these results suggest that CK delays the DIS via accumulation of HmChl, probably by regulating the conversion of Chl *a* to Chl *b* and maintaining the Chl *a*/*b* ratios and pigment–protein complexes.

In summary, we report that CK retards senescence in cut leaves of N22 following dark treatment and HmChl accumulates in response to CK treatment. Our results show that HmChl levels increase till 96h following CK treatment, thereby maintaining the Chl *a*/*b* ratio during the delay of dark-induced leaf senescence. The accumulation of HmChl appears to have an adaptive value since it is stable, has a similar molecular structure and absorption spectra to Chl *a* and Chl *b*, and is probably incorporated in the pigment–protein complexes functioning as a light-harvesting pigment as suggested by Nagane *et al.* (2010). The light energy absorbed by HmChl may be transferred to neighboring pigments without producing reactive oxygen species since *hmc1* mutants of *Arabidopsis thaliana* which accumulate HmChl do not show necrosis and cell death (Nagane *et al.*, 2010). It is known that under dark conditions, Chl *a* is irreversibly degraded to catabolites, resulting in a major decrease in levels of total Chl, breakdown of pigment–protein complexes, and yellowing of leaves (Hörtensteiner, 2006). HmChl may be serving as a stable substrate ensuring continued availability of Chl *a* which would otherwise be degraded and not be available for conversion to Chl *b*, thereby maintaining the Chl *a*/*b* ratio. We conclude that CK affects the Chl *a*/*b* interconversion cycle and maintains the stability of photosynthetic pigment complexes, resulting in prolonged greenness during senescence.

## Supplementary data

Supplementary data are available at *JXB* online.

Table S1. List of primers used in this study.

Supplementary Data

## Abbreviations:

BA: 6-benzyl adenine
Chl: chloropyll
CK: cytokinin
DIS: dark-induced senescence
HmChl: 7-hydroxymethyl chlorophyll
8HQ: 8-hydroxyquinoline
LHC: light-harvesting complex
N22: Nagina 22
PS: photosystem.
