# Supplementary material for: Cytokinin delays dark-induced senescence in rice by maintaining the chlorophyll cycle and photosynthetic complexes
Source: J Exp Bot. 2016 Jan 29;67(6):1839–51. doi: 10.1093/jxb/erv575 (PMC4783366; doi:10.1093/jxb/erv575)
Supplement: Supplementary Data [file supp_67_6_1839__index.html]

Cytokinin delays dark-induced senescence in rice by maintaining the chlorophyll cycle and photosynthetic complexes — Cytokinin delays dark-induced senescence in rice by maintaining the chlorophyll cycle and photosynthetic complexes — Supplementary Data 

# Cytokinin delays dark-induced senescence in rice by maintaining the chlorophyll cycle and photosynthetic complexes

## Supplementary Data

Data files

- supplementary\_table\_S1.pdf - Supplementary Data
