# Supplementary material for: Cytokinin delays dark-induced senescence in rice by maintaining the chlorophyll cycle and photosynthetic complexes
Source: J Exp Bot. 2016 Jan 29;67(6):1839–51. doi: 10.1093/jxb/erv575 (PMC4783366; doi:10.1093/jxb/erv575)
Supplement: Supplementary Data [file supp_erv575_supplementary_table_S1.pdf]

Cytokinin Delays Dark-Induced Senescence in Rice by Maintaining Chlorophyll Cycle and Photosynthetic Complexes. *Sai Krishna Talla, Madhusmita Panigrahy, Saivishnupriya Kappara, Nirosha P, Sarla Neelamraju, and Rajeshwari Ramanan*

Supplementary Table S1. List of primers used in this study

**Gene**	**Forward primer (5′–3′)**	**Reverse Primer (5′–3′)**
Abs	CATGACACTCACGCATCAGG	CCGGGTTGTGATGGATGTTC
Cdg	GCGTGCTGTATTTCCTCGAG	GCAGCACATCCATCCTCTTG
Rr1	CAGTGATCGTCATGTCGTCG	GCTGTACGTCCTTGCTTTGA
Rr4	AAGCTCAAGTCTCACCTGCT	TTCATCTGGTTTGTGTGGCG
Cbp	GATCTCGACGCTGATCTGGA	GTACAGCCTCTTCTCCGGGT
Oep	CCTACACCCTCGACGAGATC	GGCGACCAGATTCTTGATGG
Amt	CGACTTCATCAGGGGTCATT	GGAAGTCCGATTGTTGGAGA
Gad	CTCCGTGGTGACAGGGTACT	GATCATCAACGACGAGCTGA
Mtn	GAGTACATGCCCTTCTCCCTCT	GTAGAACACGTAGAGCCCCATC
Ppo	GGAGCACGATTTGGAAAGAG	CGCGTAGTCAGGGAGTTGAG
Hks	CTTTCCCAGCAGTTCCAGAG	GCAAGTCTCGATCCCAAGAG
Bgs	AATGTCCCGATGATGTTGGT	CGTTCTTCTTCGGCGACTAC
Inv	AGTTTAATGCCGGTTTGCAC	GCTGTGCCTCTTGATGACAA
Actin	CGGGAAATTGTGAGGGACAT	AGGAAGGCTGGAAGAGGACC
